# Supplementary material for: Tau exacerbates excitotoxic brain damage in an animal model of stroke
Source: Nat Commun. 2017 Sep 7;8:473. doi: 10.1038/s41467-017-00618-0 (PMC5589746; doi:10.1038/s41467-017-00618-0)
Supplement: Supplementary file 1 — Supplementary information [file 41467_2017_618_MOESM1_ESM.pdf]

Description of Supplementary Files

File Name: Supplementary Information

Description: Supplementary figures, supplementary tables

File Name: Supplementary Data 1

Description: Differentially regulated genes in tau-/- mice after PTZ administration

File Name: Supplementary Data 2

Description: Similarly regulated genes in tau-/- mice after PTZ administration

File Name: Peer Review File

Tau exacerbates excitotoxic brain damage in an animal model of stroke

Mian Bi, Amadeus Gladbach, Janet van Eersel, Arne Ittner, Magdalena Przybyla, Annika van Hummel, Sook Wern Chua, Julia van der Hoven, Wei S. Lee, Julius Müller, Jasneet Parmar, Georg von Jonquieres, Holly Stefen, Ernesto Guccione, Thomas Fath, Gary D. Housley, Matthias Klugmann, Yazi D. Ke & Lars M. Ittner

Supplementary Figures

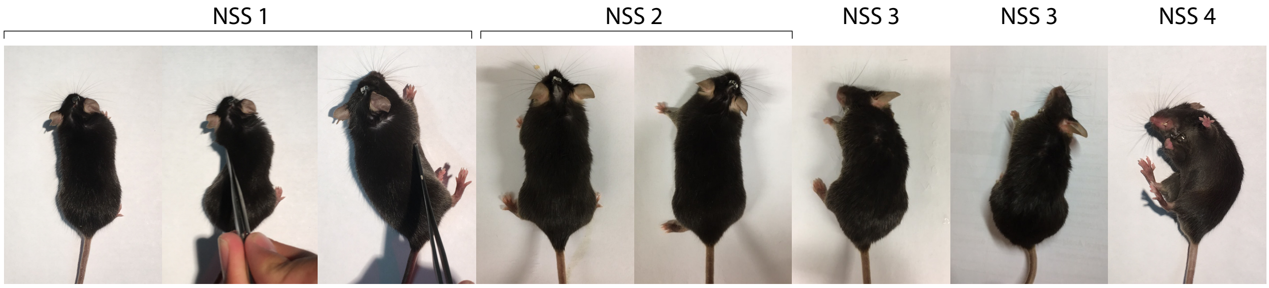

Supplementary Figure 1 Examples of mice with neurological severity scores (NSS) of '1' to '4' after MCAO. Note that mice that showed resistance to lateral push were scored as '0', even if they presented with slight asymmetry of the forelimb.

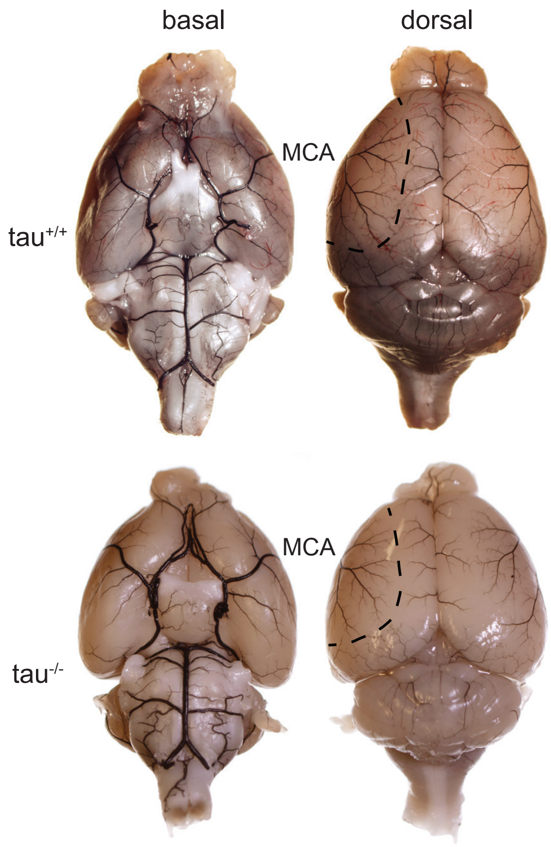

Supplementary Figure 2 Normal anatomy of the cerebral vasculature in tau$^{-/-}$ mice. Basal and dorsal aspect of tau$^{+/+}$ and tau$^{-/-}$ brains following perfusion with Indian ink/gelatin showing comparable vascular anatomy.

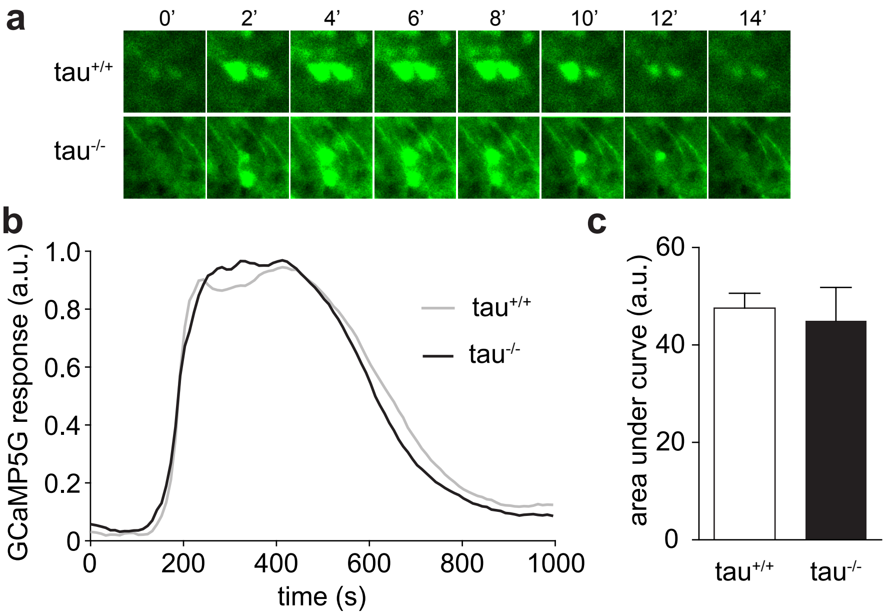

Supplementary Figure 3 Indistinguishable excitability of cortical neurons in tau$^{-/-}$ and tau$^{+/+}$ mice. (**a**) Example of temporal excitation of GCaMP5G Ca^{2+} influx reporter-expressing neurons (green) in cortical slices from tau$^{-/-}$ and tau$^{+/+}$ brains after exposure to 1mM glutamate. (**b**) Similar compound Ca^{2+} response of cortical neurons upon 1mM glutamate challenge in slices from tau$^{-/-}$ and tau$^{+/+}$ brains. (**c**) Area under the curve analysis of compound Ca^{2+} responses as shown in (B) showed no differences (not significant; N=5; Student’s t-test). Error bars are s.e.m..

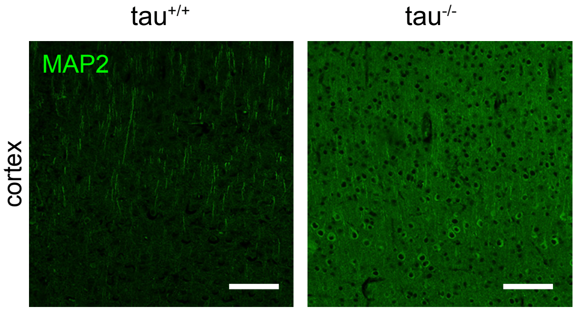

Supplementary Figure 4 No MAP2 loss following transient MCAO in tau$^{-/-}$ mice. Representative IF staining of cortex 3h after 90 minutes of transient MCAO showed loss of neuronal MAP2 in tau$^{+/+}$ but not in the corresponding brain areas of tau$^{-/-}$ mice, suggesting early neuronal damage. Scale bars, 50µm

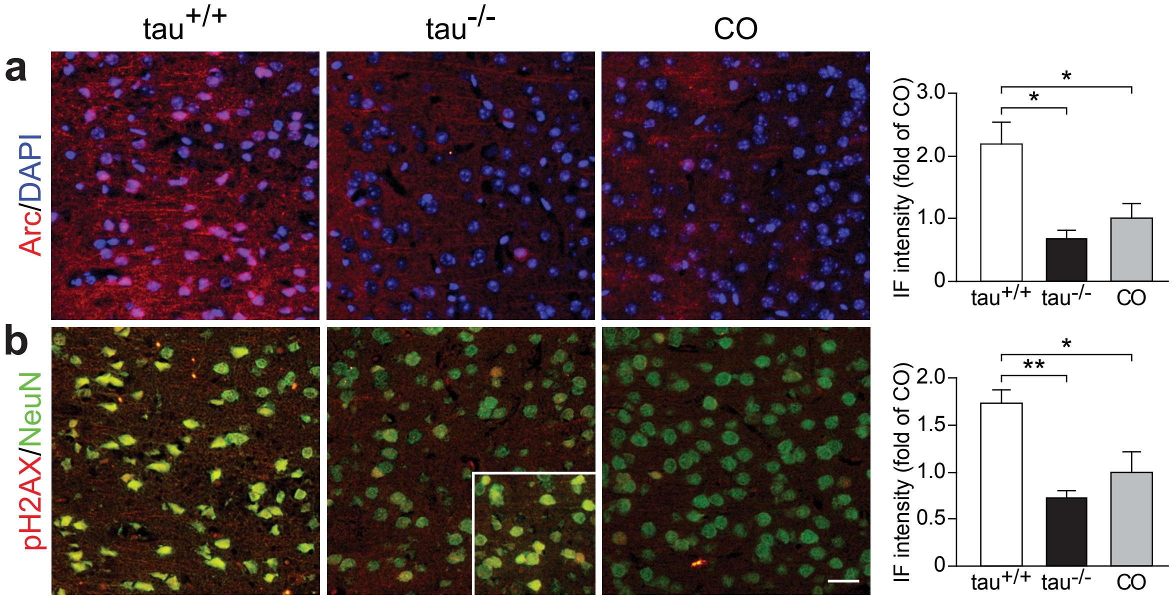

Supplementary Figure 5 Increased Arc expression and H2AX phosphorylation in tau$^{+/+}$ but not tau$^{-/-}$ mice after 90 minutes of transient MCAO. (**a**) Consistent with up-regulation of Arc mRNA 1h after transient MCAO (see Figure 4a), Arc protein is readily detectible in cortical neurons in tau$^{+/+}$ but not tau$^{-/-}$ cortex 3h after transient MCAO. Sham operated tau$^{+/+}$ controls (CO) show no Arc IF. Quantification from independent staining (*, P<0.05; N=4; 1-way ANOVA [Tukey post-hoc]). (**b**) Phosphorylation of H2AX (red) overlying (yellow) with NeuN in neurons (green) indicates cell/DNA damage in cortical neurons of tau$^{+/+}$ mice 3h after transient MCAO. No such staining was found in the comparable brain region of tau$^{-/-}$ after MCAO or sham operated CO mice. Note the pH2AX staining in the core region in tau$^{-/-}$ brains (inset), reflecting the initial ischemic infarct. Quantifications from independent staining (*, P<0.05; **, P<0.01; N=4; 1-way ANOVA [Tukey post-hoc]). All error bars are s.e.m..

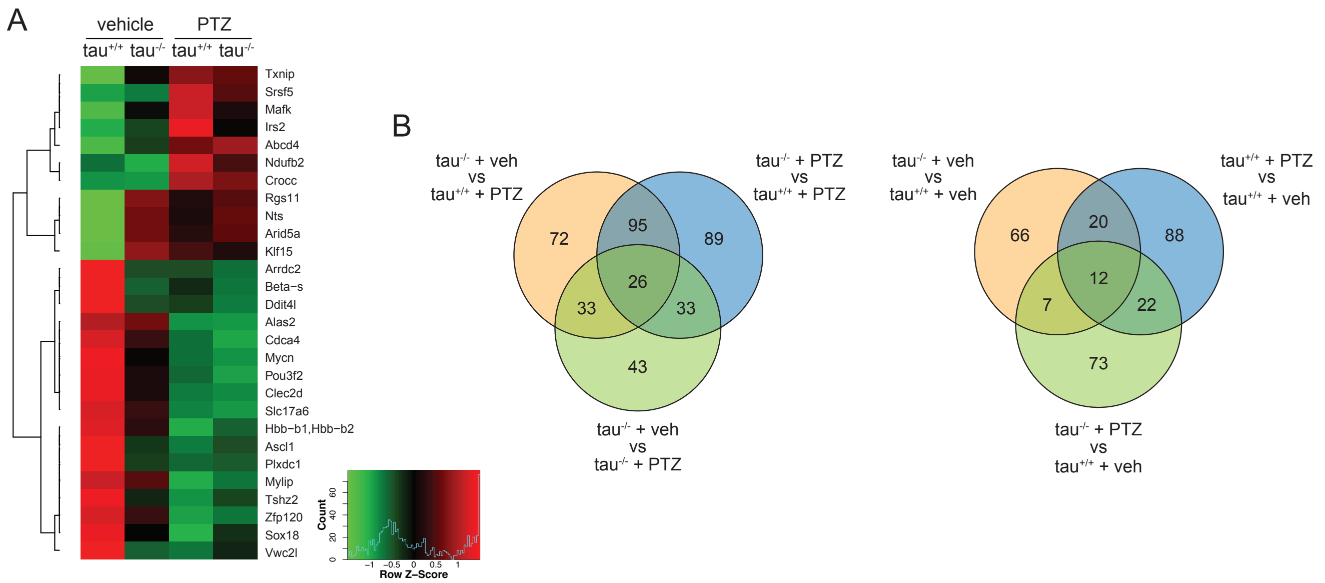

Supplementary Figure 6 Gene regulation in tau$^{+/+}$ and tau$^{-/-}$ mice with induced seizures. (**a**) Genes with similar induction or suppression in tau$^{+/+}$ and tau$^{-/-}$ mice upon PTZ administration. Whole transcriptome sequencing of tau$^{-/-}$ and tau$^{+/+}$ mice treated with vehicle or PTZ reveals similar gene expression response 1h after treatment in tau$^{-/-}$ mice. Red indicates up- and green down-regulation. Genes with significant differential regulation are displayed. Genes are listed in table S3. (**b**) Venn Diagrams showing the number of differentially expressed genes at an FDR of 5%.

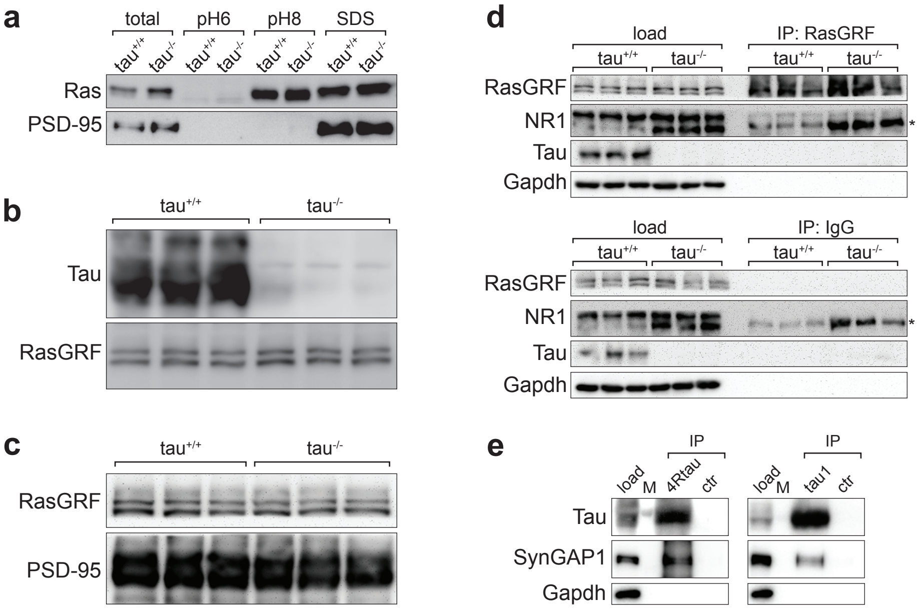

Supplementary Figure 7 Unaltered Ras and RasGRF in tau$^{-/-}$ mice. (**a**) Sequential synaptosome preparation from tau$^{+/+}$ and tau$^{-/-}$ mice showing comparable levels of synaptic Ras. PSD-95 enrichment in the post-synaptic density (SDS) fraction confirmed purity of preparations. (**b**) Levels of RasGRF were comparable in cortical brain extracts from tau$^{+/+}$ and tau$^{-/-}$ mice. Tau was only detectable in tau$^{+/+}$ mice. (**c**) RasGRF levels in the post-synaptic density (SDS) fraction of synaptosomes from tau$^{+/+}$ and tau$^{-/-}$ mice were comparable. PSD-95 confirmed the same level of enrichment in preparations from tau$^{+/+}$ and tau$^{-/-}$ mice. (**d**) Immunoprecipitation (IP) from tau$^{+/+}$ and tau$^{-/-}$ cortical brains extracts using RasGRF antibodies showed similar co-precipitation of NR1, but failed to co-precipitate tau. Co-IP with an IgG control confirmed specificity and revealed and unspecific band detected predominantly in loads and IPs from tau-/- mice (asterisk). Load confirmed equal input for IPs and Gapdh equal loading. (**e**) Additional SynGAP1/tau co-immunoprecipitation (co-IP). SynGAP1 interacts with tau in brain extracts from wild-type mice, as revealed by co-IP using 2 different tau-specific antibodies (4Rtau and tau1) in addition to the co-IP presented in the main Figure 6n. Control (ctr) precipitations were done without primary antibodies. M, marker lanes.

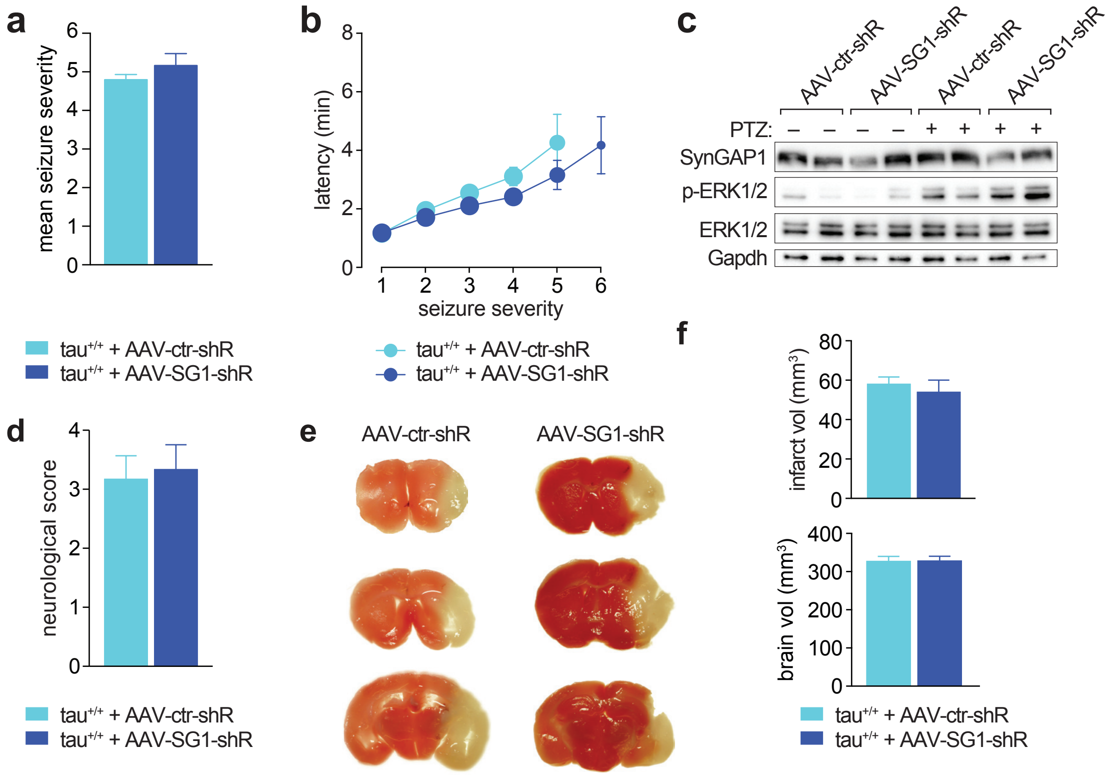

Supplementary Figure 8 Effects of SynGAP1 knockdown in tau+/+ mice. (**a**) Mean seizure severity in AAV-SG1-shR- or AAV-ctr-shR-injected tau$^{+/+}$ mice after administration of 50mg/kg PTZ (*N*=7 (AAV-ctr-shR), *N*=10 (AAV-SG1-shR)). (**b**) Latency to develop higher degree seizures after administration of 50 mg/kg PTZ in AAV-SG1-shR- or AAV-ctr-shR-injected tau$^{+/+}$ mice. Some AAV-SG1-shR-injected, but no AAV-ctr-shR-injected tau$^{+/+}$ mice reached full extension seizures (score 6) (*N*=7 (AAV-ctr-shR), *N*=10 (AAV-SG1-shR)). (**c**) More ERK1/2 phosphorylation 10 min after 50mg/kg PTZ administration in AAV-SG1-shR-injected, compared to AAV-ctr-shR-injected tau$^{+/+}$ mice, together with reduction of SynGAP1 levels. Detection of ERK1/2 and Gapdh confirmed equal loading. (**d**) Neurological scoring revealed similar deficits 1 and 24 hours after transient MCAO in AAV-SG1-shR- and AAV-ctr-shR-injected tau$^{+/+}$ mice (*N*=5). (**e**) Representative TTC-stained brain sections of tau$^{+/+-}$ mice injected with AAV-SG1-shR or AAV-ctr-shR 24h after transient MCAO (viable tissue stains red). (**f**) Volumetric quantification of serial TTC-stained sections showed similar infarct volumes in AAV-SG1-shR-injected compared to AAV-ctr-shR-injected tau$^{+/+}$ mice, together with comparable total brain volumes (*N*=5). All error bars are s.e.m..

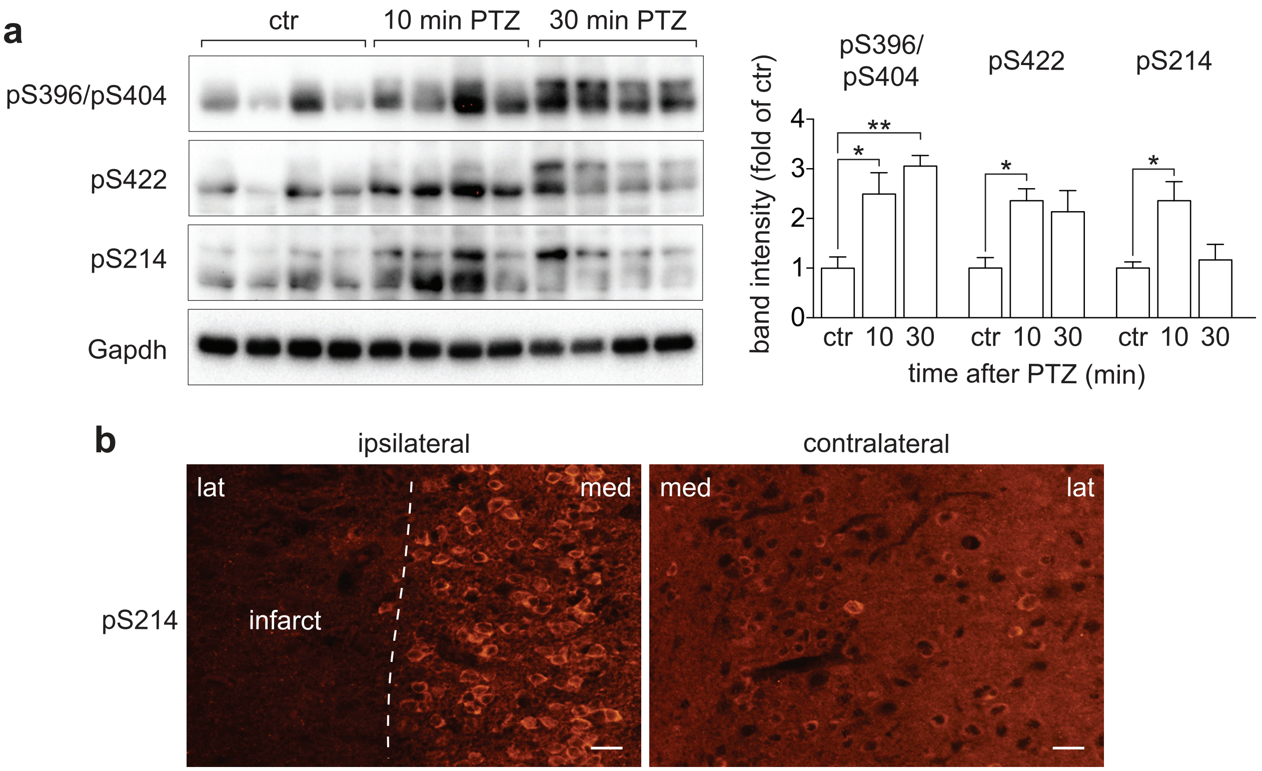

Supplementary Figure 9 Increased tau phosphorylation after PTZ and MCAO. (**a**) Western blotting of cortical brain extracts without, and 10 and 30 minutes after 50mg/kg PTZ administration. Levels of tau phosphorylation at several sites (pS214, pS396/S404 and pS422) increased after $tau^{+/+}$ mice were challenged with PTZ (*, *P*<0.05; **, *P*<0.01; *N*=4; 1-way ANOVA [Tukey post-hoc]). Detection of Gapdh confirmed equal loading. (**b**) Increased tau phosphorylation at pS214 in the perimeter of infarct areas (broken line) in the cortex 3 hours after 90 minutes of transient MCAO, as detected by immunofluorescence staining (red). Staining of the contralateral site is provided for comparison. lat, lateral; med, medial. Scale bars, 50µm. All error bars are s.e.m..

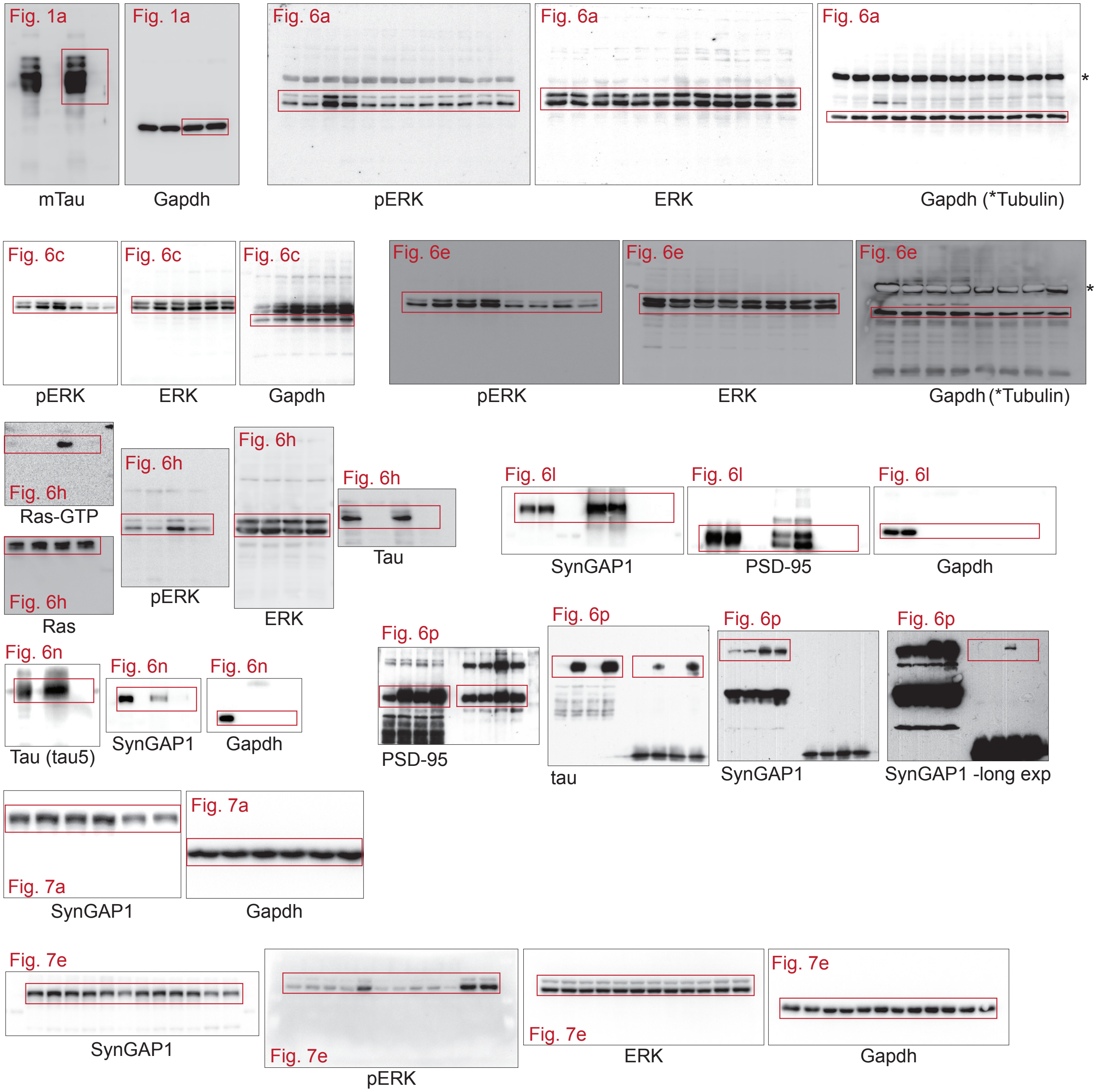

[continued on next page]

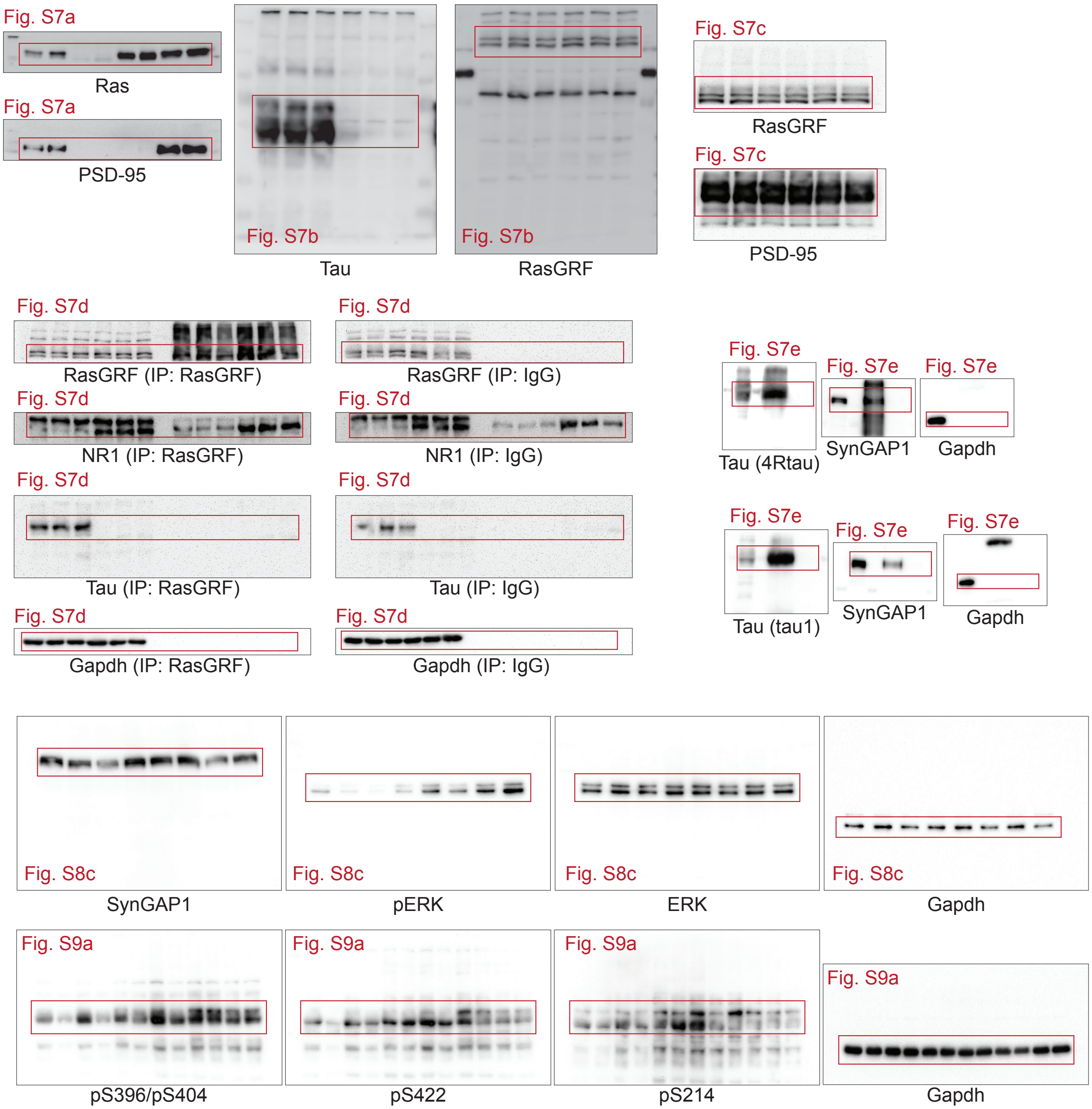

Supplementary Figure 10 Full membranes detected for Western blots shown in (red) indicated main and supplementary figures. Red boxes highlight the sections of blots shown in indicated figures. Note that some membranes were trimmed to size prior to detection to reduce antibody volume and fit incubation containers.

Supplementary Tables

Supplementary Table 1 Similar physiological parameters before and after transient MCAO in $tau^{+/+}$ (N=3) and $tau^{-/-}$ (N=3) mice. Note that there were no significant changes between groups at 0h and 1h for any of the parameters measured.

	0h			1h		
**Parameter (units)**	**$tau^{+/+}$**	**$tau^{-/-}$**	***p*-value**	**$tau^{+/+}$**	**$tau^{-/-}$**	***p*-value**
Body temperature (ºC)	35.3±0.3	35.7±0.3	0.5185	35.7±0.3	36.0±0.0	0.3739
Blood pressure–systolic (mmHg)	121.7±6.4	127.7±3.4	0.4531	110.0±8.5	118.7±3.3	0.3965
Blood pressure–diastolic (mmHg)	103.0±7.6	104.0±4.2	0.9134	91.3±7.9	95.7±2.7	0.6308
O2 saturation (%)	81.0±0.6	79.7±1.9	0.5304	84.7±5.2	89.7±1.8	0.4122
Perfusion rate	0.38±0.03	0.34±0.02	0.3661	0.37±0.03	0.31±0.02	0.1700
Heart rate (bpm)	259.0±7.2	264.7±9.4	0.6579	375.7±69.6	344.7±35.5	0.7117
pH	7.18±0.05	7.16±0.09	0.8975	7.08±0.02	7.02±0.04	0.1927
pCO2 (mmHg)	50.1±9.9	57.5±14.2	0.6927	71.1±3.2	80.1±5.2	0.2105
BEecf (mmol/Ll	-10.0±2.0	-9.3±1.2	0.7893	-8.3±0.3	-10.3±1.9	0.3486
HCO3 (mmol/L)	18.3±2.3	19.5±0.9	0.6311	21.3±0.3	20.6±1.1	0.5862
Total CO2 (mmol/L)	19.7±2.6	21.7±1.2	0.5239	23.3±0.3	23.3±1.2	>0.999
Sodium (mmol/L)	148.7±0.9	149.7±1.2	0.5391	158.0±6.0	171.7±0.3	0.0853
Potassium (mmol/L)	5.1±0.6	5.9±0.4	0.3180	5.1±0.2	5.4±0.1	0.1700
Calcium (mmol/L)	1.22±0.02	1.24±0.02	0.8512	1.30±0.07	1.52±0.06	0.0636
Hematocrit	0.46±0.03	0.47±0.01	0.6401	0.42±0.003	0.42±0.01	0.7676

Supplementary Table 2 KEGG pathway analysis of genes that lack induction in PTZ-injected $tau^{-/-}$ mice.

**KEGG Pathway**	**Count**	***p*-value**	**Genes**
MAPK	12/265	0.00001	DUSP5, FOS, DUSP4, BDNF, DUSP1, JUN, GADD45G, NR4A1, GADD45B, SRF, CD14, DUSP6
p53	4/69	0.0229	CDKN1A, GADD45G, THBS1, GADD45B
Toll-like receptor	4/99	0.0572	FOS, JUN, LBP, CD14
Wnt	2/149	0.0999	WNT10A, WNT9A
Melanogenesis	2/100	0.0679	WNT10A, WNT9A
Basal cell carcinoma	2/55	0.0378	WNT10A, WNT9A
Hedgehog	2/54	0.0371	WNT10A, WNT9A

Supplementary Table 3 Primers for quantitative PCR analysis.

Gene name	Forward Primer (5'→3')	Reverse Primer (5'→3')
Arc	GTGTGGAGGGAGGTCTTCT	CTGCCCACTGGGTATTTGC
Fos	TACTACCATTCCCCAGCCGAC	TTGGCACTAGAGACGGACAGA
JunB	GGCCACCAAGTGCCGGAA	CGTTCTCAGCCTTGAGTGT
Egr1	AGGGGAGCCGAGCGAACA	CGCCTTCTCATTATTCAGAGC
c-jun	ATCGCTCGGCTAGAGGAAA	ACCTGTTCCCTGAGCATGT
FosB	TCCAGCCAGAGCCAGGC	CTCTGCGAACCCTTCGCTT
Fosl2	TGATCAAGACCATCGGTAC	TCGACGCTTCTCCTCCTC
Ptgs2	TGGGGTGATGAGCAACTATT	CACGTAGTCTTCGATCACTA
Mapt	TCGGAGACCTCCGATGCT	TTGTCGGGAGCTCTCTCATC
Mmp16	GGGAAGATGTTGGCAAAACC	AGGGTCCATGGTTTTCATTTC
Crocc	GCCCAGCGCCTACGCAGT	CTGGGTCACCTGCAGGCTG
Srsf5	CCGAGATATTGACTTGAAAAG	ATAAACAGCATCATCTGCATC
Dusp5	CCCGTTCTCAGCGTCGCC	TCCAAGGTAGAGGAAGGGAA
Zfp2	GAATCTTACAGTGCACCAGAG	TGCTTTGGCTGAAGGCTTTG
Cdca4	GAGACCCTGGAGAACAAAAAC	TGTGTCCAGGTCATAGTAGGA
Actin	CCCCCTGAACCCCAAAGC	CCACGTACATGGCTGGGGT
GAPDH	GTGAAGGTCGGTGTGAAC	ATCTCCACTTTGCCACTGCAA
